# The Lone Star Tick, Amblyomma americanum, salivary factors exacerbate the clinical outcome of Heartland virus disease in a small animal model

**DOI:** 10.21203/rs.3.rs-2828801/v1

**Published:** 2023-04-25

**Authors:** Erin S. Reynolds, Jacob T. Wooldridge, Heather Stevenson, Saravanan Thangamani

**Affiliations:** Upstate Medical University; University of Arkansas for Medical Sciences; University of Texas Medical Branch; Upstate Medical University

## Abstract

Heartland virus was first isolated in 2009 from two patients in Missouri and is transmitted by the lone star tick, *Amblyomma americanum.* To understand disease transmission and pathogenesis, it is necessary to develop an animal model that utilizes the natural transmission route and manifests in a manner similar to documented human cases. Herein we describe our investigations on identifying A129 mice as the most appropriate small animal model for HRTV pathogenesis that mimics human clinical outcomes. We further investigated the impact of tick saliva in enhancing pathogen transmission and clinical outcomes. Our investigations revealed an increase in viral load in the groups of mice that received both virus and tick salivary gland extract (SGE). Spleens of all infected mice showed extramedullary hematopoiesis (EH), depleted white pulp, and absence of germinal centers. This observation mimics the splenomegaly observed in natural human cases. In the group that received both HRTV and tick SGE, the clinical outcome of HRTV infection was exacerbated compared to HRTV-only infection. EH scores and viral antigens in the spleen were higher in mice that received both HRTV and tick SGE. In conclusion, we have developed a small animal model that mimics natural human infection and also demonstrated the impact to tick salivary factors in exacerbating the HRTV infection.

## Background

**Figure 1:**
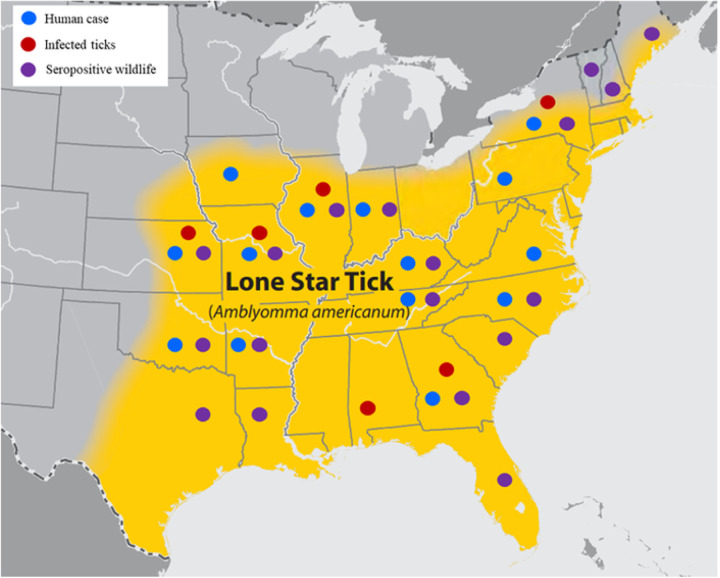
Known distribution of *Amblyomma americanum* and Heartland virus. Current CDC estimates for *A. Americanum* distribution (https://www.cdc.gov/ticks/geographic_distribution.html) overlayed with the known occurrence of human cases (blue dot), infected ticks (red dot), and seropositive wildlife (purple dot).

Ticks have been a known vector for viruses that cause human disease for over a century (1), and the geographic distribution of ticks and the pathogens they transmit spans much of the globe (2, 3). Although the reasons are not fully understood, the number of cases of tick-borne viruses has been increasing over the last few decades (3–7). Since 2007, newly discovered tick-borne viruses, including Dabie bandavirus, the causative agent of Severe Fever and Thrombocytopenia Syndrome (SFTS), Bourbon virus, the causative agent of Bourbon virus disease, and Heartland virus (HRTV), the causative agent of Heartland virus disease, have been identified by both human cases and retrospective examination of human or animal serum and field-collected ticks (8–11). Heartland virus (family *Phenuiviridae*, genus *Bandavirus*) is an emerging tick-borne disease, first isolated in 2009 from two ill humans in Missouri, USA (9). Following the initial discovery of HRTV, there have been over 50 confirmed human cases spanning 14 states (Fig. 1) (12, 13). Symptoms of HRTV disease are similar to the closely related SFTS, which was isolated in China in 2007 (8), and include fever, headache, fatigue, nausea, muscle and joint pain, and anorexia, with blood work showing thrombocytopenia, leukopenia, and elevated liver enzymes (9, 12, 14). There have been four known fatal cases of HRTV, all in individuals with substantial comorbidities (14–17). Screening of blood collected in 2013 from blood donors in northwestern Missouri estimated human prevalence of HRTV disease in endemic areas at 0.9%, while testing individuals with the disease of unknown etiology and symptoms consistent with HRTV disease identified acute and previous infection across seven states (18, 19). The Lone Star tick, *Amblyomma americanum*, has been implicated as the vector for HRTV. Pools of field collected *A. americanum* ticks caught adjacent to the homes of the index patients, as well as in nearby regions of Missouri and Kansas, have tested positive for HRTV (20–23). Lone star ticks are aggressive in host-seeking and readily feed on humans (24–26). The current range of *A. americanum* includes much of the Southeast, Northeast, and Midwest United States, but reports of geographic expansion, as well as modeling of climate change scenarios, indicate increased areas where environmental conditions are suitable for the establishment of *A. americanum* (27–31). Although human cases of HRTV have not been detected in all areas where *A. americanum* is present, limited surveillance of wild and domestic animals in these areas have demonstrated seropositivity in multiple species including white-tailed deer (*Odocoileus virginianus*), raccoons (*Procyon lotor*), coyotes (*Canis latrans*), and moose (*Alces alces*) in 18 states and HRTV infected ticks in six states (Fig. 1) (21, 23, 32–37). Efforts to develop suitable animal models to study HRTV transmission and pathogenesis have been limited and immunocompetent animals do not recapitulate symptoms of human disease (38–40). In both previously published work and based on our experiments, only animals with Interferon (IFN) deficiencies appear susceptible to HRTV infection (38, 41, 42). To date, animal model development efforts have focused primarily on the interactions between pathogen and host without thoroughly examining the arthropod vector’s role in disease transmission and pathogenesis. The feeding process of Ixodid ticks is very different from other blood-feeding arthropods in regard to the amount of blood collected and the duration of the feeding. While the bite of a mosquito, flea, or midge can be completed within a few minutes, Ixodid ticks feed for several days or weeks and significantly increase in size to accommodate a large blood meal. To facilitate feeding and avoid detection, tick saliva has evolved a complex combination of pharmacologically active compounds that assist in blocking, modulation, and suppression of the host the itch and pain response, immune response, wound healing, and coagulation responses (43, 44). These compounds have been shown to change throughout the feeding process and may vary between tick life stages (45, 46). While the purpose of these compounds benefit the vector, they also create an environment permissive to pathogen transmission through a process called saliva-activated transmission (SAT) and exacerbate the disease course (43, 47–49). To understand the transmission and pathogenesis of arthropod-borne viruses, it is essential to examine the interactions between pathogen, host, and vector.

## Materials And Methods

### Ethics Statement.

Animal experiments were conducted in compliance with the guidelines of the Association for Assessment and Accreditation of Laboratory Animal Care International (AAALAC) in an AAALAC-approved facility. Study protocols (1504024 and 0112059) were approved by the University of Texas Medical Branch (UTMB) Institutional Animal Care and Use Committee (IACUC.) Following arrival or transfer to UTMB Animal Biosafety Level 3 (ABSL3) facilities animals were allowed to acclimate to the facility prior to study initiation. The data described in this manuscript are in accordance with ARRIVE guidelines.

### Tick Salivary Gland Extract.

Adult *Amblyomma americanum* ticks which were free of known human pathogens were purchased from the Oklahoma State University Department of Entomology and were allowed to feed on New Zealand White rabbits. Approximately two weeks post attachment (13–15 days) but before completion of feeding, one male and four engorged female ticks were removed. Salivary glands were dissected, pooled, and stored in 300 µL Phosphate Buffered Saline (PBS) (Corning) at −80°C. Prior to the experimental study, the pooled salivary gland extract (SGE) was thawed over wet ice and homogenized in a TissueLyser II (Qiagen.) Based on the total volume of the suspension each animal received 15 µL of SGE, which was approximately half of one salivary gland.

### Virus.

Heartland Virus strain MO-4, which was isolated from the human index case (9), was provided by the Work Reference Center for Emerging Viruses and Arboviruses (WRCEVA) (UTMB). The virus was passaged four times on Vero E6 cells (CRL-1586) (American Type Culture Collection.) The virus titer was determined via a focus-forming assay (FFA) adapted from previously published methods (50, 51). Vero E6 cells were seeded onto 12-well plates (Corning) and allowed to adhere overnight. A culture medium of Dulbecco’s Modified Eagle Medium (DMEM) (Gibco) supplemented with 2% Fetal Bovine Serum (Hyclone) and 1% Penicillin-Streptomycin (Corning) were supplied, and plates were maintained at approximately 37°C with 5% CO_2_. Immediately prior to infection, media was aspirated from the plates, and serial dilutions of HRTV were dispersed onto the cell monolayers in triplicate. One column of wells was utilized as a negative control and had media dispensed onto it. Plates were incubated for one hour and gently rocked every 10 to 15 minutes to ensure virus distribution. Following incubation, 0.8% methylcellulose overlay was added, and plates were maintained for four days. Methylcellulose was removed, and plates were fixed with methanol: acetone (1:1). Plates were allowed to air dry completely. Cell membranes were permeabilized by incubating with 0.5% Triton X-100 (Sigma-Aldrich) in PBS for five minutes and then washed with 0.05% Tween20 (Sigma-Aldrich) in PBS (PBST). A blocking solution of PBST with 5% goat serum (Sigma-Aldrich) and 1% bovine serum albumin (Fisher Scientific) was incubated on wells for one hour, followed by an additional hour incubation of mouse immune ascitic fluid against HRTV strain MO-4 (1:500) (WRCEVA). Plates were washed with PBST prior to a one-hour incubation with goat anti-mouse IgG secondary antibody conjugated to horseradish peroxidase (1:1000) (Invitrogen). Following another PBST wash, AEC substrate prepared and used in accordance with the ImmPACT AEC Peroxidase (HRP) Substrate kit (Vector Labs) was added to each well and allowed to develop in the dark.

### Host Susceptibility Study.

Initial experiments were conducted to assess the susceptibility of various strains of laboratory mice to HRTV infection by intraperitoneal (IP) and footpad (FP) injection. All mice received a single injection of 1.49×10^5 FFU HRTV and were monitored up to 18 days post-infection (dpi). Uninfected control groups were utilized for each strain of mouse infected with HRTV. Control mice were grouped in the number, sex, strain, and age as experimentally infected mice but received a single IP injection of PBS instead of HRTV. These mice were handled in the same manner and underwent the same procedures as experimentally infected mice. Immunocompetent CD-1 mice and C57BL/6J, purchased from Charles River Laboratories (Wilmington, MA) and The Jackson Laboratory (Bar Harbor, ME), respectively, were first assessed. Both strains of mice were approximately seven weeks old at study initiation. Following the assessment of immunocompetent mouse strains, two IFN-deficient strains of mice were evaluated. STAT1 knock-out mice, which are IFN-α/γ deficient, were purchased from Taconic Biosciences (Germantown, NY) and A129 knock-out mice, which are IFN α/β deficient, were bred and maintained in the specific pathogen free colony facility at UTMB. STAT1 mice were approximately five weeks old at study initiation, and A129 mice were five to six weeks old at study initiation.

Following infection all mice were monitored for clinical manifestation of the disease (Supplementary Table S1), whole blood was collected by retro-orbital sinus prior to infection and every two to three days thereafter to evaluate viremia, and at study termination, whole blood was collected by cardiac puncture and the spleen, liver, kidneys, and muscle adjacent to the injection site were collected to determine viral load. Mice were sedated by isoflurane inhalation for FP injection and retro-orbital sinus blood collection. Euthanasia was performed by CO_2_ inhalation followed by cervical dislocation. Blood and tissue samples were stored in either TRIzol LS or TRIzol (ThermoFisher Scientific) until homogenized for RNA extraction and subsequent qualitative rt-PCR analysis.

### Impact of Tick Saliva on HRTV Pathogenesis.

Eighteen A129 mice, 10 males and 8 females were used to evaluate the effects of tick saliva on HRTV pathogenesis. At study initiation three female mice aged 33 days and one female mouse aged 22 days were designated as uninfected controls. The remaining 10 male and four female mice, all aged 22 days, were weighed and designated to receive HRTV-only or HRTV plus SGE. The mean weight for these groups was 9.32 g and 9.18 g, respectively. All mice received a single injection of 45 µL in the right hind footpad. Negative control mice received culture medium, the virus only mice received 1.35×10^5^ FFU HRTV, and virus plus SGE mice received 1.35×10^5^ FFU HRTV combined with SGE. Monitoring for clinical disease and blood sampling were completed as described above. Additional whole blood samples were collected at euthanasia for hematology and clinical chemistry analysis. Mice were euthanized at either 3 or 8 dpi, and necropsy was immediately performed. Spleen, liver, kidneys, brain, heart, lungs, stomach, small intestine, and testes, where applicable, were subdivided and stored in TRIzol for RNA extraction or 10% Neutral Buffered Formalin (Fisher Scientific) for histopathological analysis.

### Hematology.

Prior to infection and at euthanasia whole blood was collected into tubes containing K3 EDTA and gently inverted until mixed. Samples were analyzed on the date of collection by the Hemavet 950FS instrument (Drew Scientific). The following parameters were evaluated: red and white blood cell counts; percent and counts of leukocytes; hemoglobin; hematocrit; mean corpuscular volume; mean corpuscular hemoglobin; mean corpuscular hemoglobin concentration; and platelets.

### Clinical Chemistry.

At euthanasia, whole blood was collected into serum separator tubes and analyzed on the date of collection. Samples were allowed to clot for at least 20 minutes before being centrifuged for 10 minutes at 1500xg. The analysis was completed by Vetscan VS2 instrument (Abaxis) utilizing Preventive Care Profile Plus Rotors. Parameters evaluated were blood urea nitrogen, creatinine, alanine aminotransferase, alkaline phosphatase, aspartate aminotransferase, total bilirubin, glucose, calcium, total protein, albumin, globulin, sodium, potassium, chloride, and total carbon dioxide.

### RNA Extraction.

Total RNA was extracted from tissue and whole blood samples by RNeasy Mini Kits (Qiagen, LLC) in accordance with previously published methods (52) which were modified from the TRIzol Reagent User Guide and RNeasy Mini Handbook. Immediately following collection, tissue and whole blood samples were stored in TRIzol or TRIzol LS reagent, respectively. Samples were stored refrigerated overnight to allow pathogen inactivation prior to being transferred to −80°C until RNA extraction was completed. Prior to extraction, tissues were homogenized by TissueLyser II (Qiagen). Whole blood samples were not homogenized. RNA quantity and purity were assessed by NanoDrop 1000 (Fisher Scientific) or DS-11+ (DeNovix) spectrophotometer.

### Pathogen Detection and Quantification by Real-Time Polymerase Chain Reaction.

Quantitative real-time polymerase chain reaction (qRT-PCR) was completed by combining the components of iTaq Universal Probes One-Step Kits (Bio-Rad) and HRTV specific primer-probe (20). This mix was dispensed onto iQ 96-Well PCR Plates (Bio-Rad), and up to one microgram of RNA was added. Plates were sealed and qRT-PCR was run on an iQ5 Real-Time PCR system (Bio-Rad) in accordance with the iTaq Universal Probes One-Step Kit protocol. To quantify viral load, RNA was extracted from two samples of known infectivity. The resulting RNA was serially diluted in duplicate, and qRT-PCR was conducted with the same materials and protocols used for experimental samples. The resulting threshold cycle (C_T_) values were averaged for each dilution and plotted with the log of the viral concentration to generate a linear equation and determine the standard curve.

### Histopathology and Immunohistochemistry.

Tissues were formalin-fixed in accordance with UTMB standard operating procedures for BSL-3 agents prior to transfer to BSL-2 laboratories. Tissues were paraffin-embedded and sectioned at 5 µm thickness. Hematoxylin-eosin staining was completed on all organs collected, and immunohistochemistry for HRTV antigen was completed for spleens adapted from previously published methods (53). Slides were deparaffinized and rehydrated in xylenes and decreasing concentrations of ethanol. Slides were submerged in DAKO Target Retrieval Solution (Agilent) and microwave heated for 20 minutes. After cooling to room temperature, slides were then incubated for five minutes while protected from light in 3% H_2_O_2_ to quench endogenous peroxidase. Mouse-On-Mouse kit (MOM) (Vector Labs) was utilized to reduce high background staining caused when mouse primary antibodies are used on mouse tissues. Slides were incubated for one hour with MOM Mouse IgG Blocking Reagent. Mouse anti-HRTV monoclonal antibody (MAb) 2AG9 (54) diluted 1:250 in MOM diluent and incubated overnight at 4°C. The secondary antibody, MOM biotinylated anti-mouse IgG diluted 1:250 in MOM working solution, was incubated for 20 minutes at room temperature. Incubation with Streptavidin-peroxidase Ultrasensitive Polymer (Sigma-Aldrich) followed by ImmPACT AEC Peroxidase (HRP) kit (Vector Labs) was completed in accordance to vendor-supplied protocols to detect biotinylated antibody. Slides were counterstained with Harris hematoxylin (Fisher Scientific) and mounted in Vectamount AQ Aqueous Mounting Medium (Vector Labs).

### Statistics.

Statistical analyses were performed using GraphPad Prism 9 (GraphPad). Data for each group were averaged and the standard error of the mean (SEM) was calculated (95% CI). Statistical significance was determined for results that had a p-value < 0.05. Viremia and viral load data were evaluated using a two-tailed, unpaired t-test with Welch’s correction. Hematology data were evaluated using Brown-Forsythe and Welch’s ANOVA test with Dunnett’s T3 multiple comparisons test.

## Results

### Host Susceptibility Evaluation.

**Figure 2:**
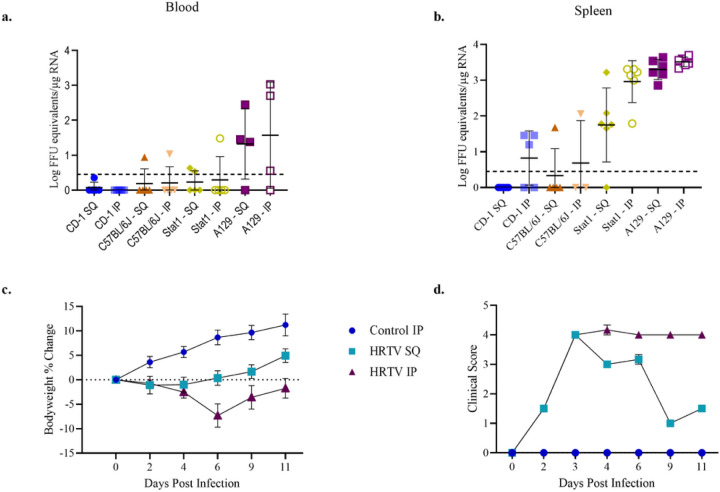
Viral RNA detection in blood and signs of disease in host susceptibility evaluation experiments. HRTV viral RNA detected in blood (a) and spleen (b) across mouse strains and route of infection. Bodyweight changes (c) and clinical score (d) of A129 mice following challenge with HRTV. Error bars show SEM.

The uninfected control mice utilized for each experiment did not develop any clinical symptoms, weight loss, or injection site complications. Immunocompetent CD-1 and C57Bl/6J mice were refractory to HRTV when infected by FP and IP injection. All mice used for these experiments were female. Five CD-1 mice were inoculated by each route. Five C57BL/6J mice were inoculated by each route, but two mice in the IP group died following inoculation. Clinical observations and body weight measurements were performed following the infection, but no signs of illness or weight loss developed. Blood was collected at 2, 4, 6, 8, 10, 12, and 18 dpi. One CD-1 mouse, which had been inoculated via the FP had detectable viremia at 10 and 18 dpi (Fig. 2). One C57BL/6J mouse inoculated via the IP route had detectable viremia at 6 dpi while one C57BL/6J mouse inoculated via the FP had detectable viremia at 12 dpi (Fig. 2). Viremia was not detected in any other CD-1 or C57BL/6J mice. At 18 dpi, all surviving mice were euthanized via CO_2_ inhalation followed by cervical dislocation. Necropsy was performed on all mice to collect the spleen, kidney, liver, and muscle adjacent to the injection site. There were no gross pathology observations. Organs were subdivided and placed in either Trizol or 10% formalin. Viral detection by qRT-PCR indicated viral RNA was present in three spleens for the IP infected CD-1 mice, one spleen for the IP infected C57BL/6J mice, and one spleen for the FP infected C57BL/6J mice (Fig. 2). The FP inoculated C57BL/6J mouse, which was viremic at 12 dpi, was the same mouse with the virus detected in the spleen. No other mice had both viremia and virus detected in the spleen.

Interferon α/γ deficient Stat1 mice had increased susceptibility to HRTV as compared to immunocompetent mice. Groups of six female mice were injected with HRTV via the FP or IP route. No clinical symptoms of disease or weight loss were observed. Blood was collected on 2, 4, 7, 9, 11, and 18 dpi. At 2 dpi, three IP injected mice and one FP injected mouse had detectable viremia (Fig. 2). One additional FP injected mouse exhibited viremia at 9 dpi. Necropsy was performed at 18 dpi, and there were no gross pathology observations. Viral RNA was detected by qRT-PCR in the spleens of all IP injected Stat1 mice and all but one spleen of FP injected mice (Fig. 2). Viral RNA was also detected in the liver of one IP injected mouse which had also been viremic at 2 dpi.

Interferon α/β deficient A129 mice were susceptible to HRTV when injected via the FP and IP route. Groups of six mice (three males and three females per group) were inoculated on day 0 and monitored until 11 dpi. Clinical symptoms developed at 2 dpi and peaked between 3 and 4 dpi for both infected groups (Fig. 2). Initially, mice presented with reduced grooming and progressed to a dull or rough coat and decreased cage activity that was normal when provoked. Beginning at 4 dpi the clinical symptoms of the FP infected mice began to decrease in severity, but the mice did not return to normalcy. Clinical symptoms of the IP infected mice did not increase or decrease in severity after 4 dpi.

At 2 dpi infected mice also began to have weight loss (Fig. 2). This was not consistent between routes of infection or within groups. Due to the natural range weight ranges between mice, weight changes were evaluated by comparing the percent change for each animal from their day 0 baseline weight. The FP infected mice had a peak weight loss at 2 dpi with an average loss of 1.1%, individual weight changes ranged from a 7.5% loss to a 5.1% gain. By 6 dpi the group average weight change was greater than baseline weights. Outside of the one 7.5% weight loss at 2 dpi, no other measurement reflected a ≥ 5% weight loss and two of the three male mice in this group did not have any weight loss. Weight loss for the IP infected mice peaked at 6 dpi with an average loss of 7.3%; individual weight changes ranged from a 15.7% loss to a 2.2% gain. All mice in this group exhibited weight loss during the experiment, and although one male mouse had a maximum weight loss of 1.7%, all other mice in the group had a loss of > 5%. By 11 dpi all mice but one female had begun gaining weight. Only two of the five mice gaining weight had reached weights higher than the baseline values.

Blood was collected at 2, 4, 6, and 11 dpi and tested for viral RNA by qRT-PCR. One female mouse in the FP infection group did not become viremic. All other infected mice had viremia during at least one collection between 2 and 6 dpi (Fig. 2). By 11 dpi no viremia was present. At necropsy, the spleen, kidney, liver, and muscle adjacent to the injection site were collected to viral load analysis and histopathology. Viral RNA was found in spleens from all infected mice by qRT-PCR.

Host susceptibility studies conducted here and previously described have demonstrated that many species of immunocompetent animals, including CD-1 and C57Bl/6 mice, hamsters, rabbits, chickens, goats, and raccoons, are refractory to HRTV infection (38). Mice and hamsters with interferon deficiency have been shown to have varying degrees of susceptibility to HRTV (38, 41, 42). Five-week-old IFN-α/γ deficient Stat1 mice did not develop clinical signs of illness but may become viremic and viral RNA has been isolated in the spleens of most infected animals. Stat2 hamsters aged four to five weeks and A129 mice aged five to six weeks, both IFN-α/β deficient, have been shown to manifest clinical symptoms of disease, and develop viremia, and viral RNA has been isolated in organs. Mortality occurred in a small percentage of Stat2 hamsters (41). Three-week-old IFN-α/β/γ deficient AG129 mice were highly susceptible to HRTV infection and have been shown to develop a severe clinical disease similar to fatal human cases (38). Despite the similarity to severe human cases, the calculated 50% lethal dose (LD50) in AG129 mice was 9 plaque-forming units (PFU) (38). Seven to 11 week old IFNAR^−/−^ mice developed severe clinical disease in a dose-dependent manner when injected subcutaneously, intraperitoneally, or intravenously (42). Based on the low calculated LD50 for AG129 mice and the results from experiments utilizing IFN-α/β deficient animals, it was determined that three-week-old A129 mice would be the optimal model to investigate salivary factors of the arthropod vector, *A. americanum*, to exacerbate HRTV disease.

### Tick saliva exacerbates Heartland virus clinical disease in A129 mice.

**Figure 3:**
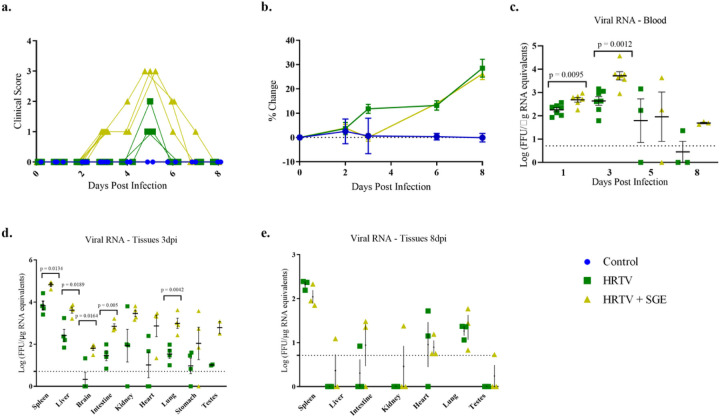
Clinical signs of disease and viral RNA detection in the impact of tick saliva on HRTV pathogenesis experiment. Animals were observed and weighed daily to assess disease and assigned a cumulative score based on the scoring table in S1 Table. Individual clinical scores and body weight changes from baseline are shown (a, b). Blood was collected at intervals post-infection, and tissues were collected at termination to determine viral load by qRT-PCR (c, d, e). Viral load data are expressed as FFU equivalents per microgram of RNA following normalization to a standard curve. Statistical significance between infected groups was determined using an unpaired two-tailed t-test with Welch’s correction. Error bars show SEM.

All mice received a single injection of 45 µL in the right hind FP. Uninfected control mice did not develop any reactions at the injection site (Fig. 3.) Two mice that received HRTV-only began developing slight swelling of the hind foot on 1 dpi which expanded to include all mice between 3 and 4 dpi. Swelling increased between 5 and 6 dpi, with one mouse having reduced usage of the right hind limb. These reactions began to resolve at 7 dpi, with only one mouse continuing to have slight swelling until study termination at 8 dpi. In contrast to the HRTV-only group, at 1 dpi, mice which received HRTV + SGE had bruising and moderate swelling of the hind foot. By 3 dpi, the swelling had progressed to include the right hind leg, and all mice had reduced usage of the effected limb. The swelling had not reduced by study termination, but two mice did regain full use of the right hind limb. At 3 dpi, mice in the HRTV + SGE group began to have reduced grooming, and clinical signs of disease peaked for this group at 5 dpi when presented with a dull or rough coat and reduced activity that became normal when provoked. Symptoms resolved for these animals between 7 and 8 dpi. Clinical symptoms were only observed in the HRTV-only group at 5 dpi, where mice had reduced grooming. The injection site reactions and clinical symptoms were more severe in the HRTV + SGE animals as compared to the HRTV-only animals. Additionally, the onset of symptoms occurred earlier and had a longer duration in the HRTV + SGE animals.

Blood was collected at 1, 3, 5, and 8 dpi to assess viremia. All infected mice became viremic, which was significantly higher in the HRTV + SGE group than in the HRTV-only group at 3 dpi (Fig. 3). The virus also appeared to persist longer in the blood of HRTV + SGE mice. At 8 dpi one of three HRTV-only mice still had detectable virus but three of three HRTV + SGE mice had detectable virus. Mice in the HRTV + SGE group also had significantly higher viral loads in the spleen, liver, brain, intestine, lung, and testes at 3 dpi than HRTV-only mice (Fig. 3). Unlike in the host susceptibility experiments with A129 mice, splenomegaly was observed in all infected mice at both necropsy timepoints. This may be due to younger mice being used for experiments utilizing tick saliva. Organ weights were not collected, but based on observations and photos taken during necropsy, as well as slides of the spleens, it appears that splenomegaly was less in the HRTV-only group.

**Figure 4:**
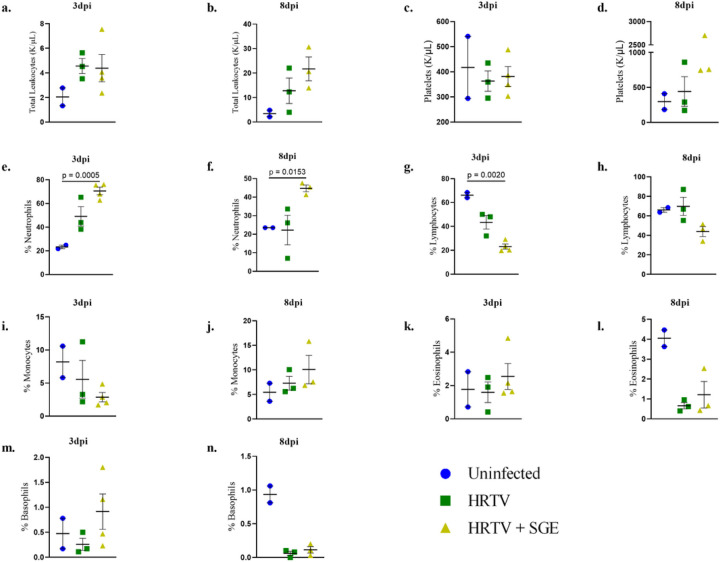
Hematology Hematology parameters were evaluated on whole blood collected at 3dpi and 8dpi terminations. Parameters evaluated included total leukocytes (a, b), platelets (c, d), and percentages of neutrophils (e, f), lymphocytes (g, h), monocytes (i, j), eosinophils (k, l), and basophils (m, n). Statistical analysis was performed using Brown-Forsythe and Welch’s ANOVA test with Dunnett’s T3 multiple comparisons test. Error bars show SEM.

Blood collected prior to infection and at necropsy for hematology analysis showed drastic changes from baseline levels at both 3 and 8 dpi for both infected groups (Fig. 4). Human cases of HRTV are characterized by thrombocytopenia and leukopenia (9, 14, 16), but these were not observed in A129 mice. Platelet counts remained stable between baseline and 3 dpi measurements and increased in the HRTV + SGE group at 8 dpi (Fig. 4, Supplementary Table S2). Both infected groups of mice had white blood cell counts that were mildly elevated at 3 dpi and much more elevated at 8 dpi. This was much more pronounced in the HRTV + SGE group. Despite the increased white blood cell counts, both infected groups had depleted lymphocytes at 3 dpi, and in the HRTV + SGE group, these values had not returned to baseline levels at 8 dpi (Fig. 4). Both infected groups also demonstrated increased neutrophils following infection, with more significant increases in the HRTV + SGE group at both 3 and 8 dpi (Fig. 4). Blood collected at necropsy for clinical chemistry was inconclusive (Supplementary Table S3).

**Figure 5:**
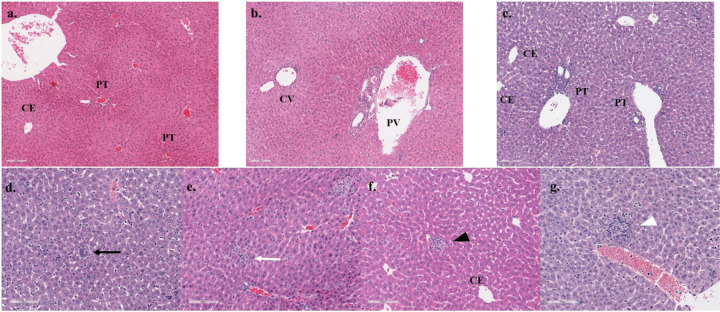
Liver pathology Hematoxylin and eosin staining of liver sections showed several histopathologic changes in infected mice. Control histology from an uninfected mouse with portal tract (PT) and central vein (CE) is shown (a). Pathology from infected mice included portal vein inflammation with portal and central venulitis (PV, CV) (b), neutrophilic inflammation of the portal tract (c), lobular megakaryocytes (black arrow, d), lobular inflammation (white arrow, e), and granuloma-like lobular lesions with eosinophilic infiltrates (black arrowhead, f and white arrowhead, g). Comprehensive pathology observations are available in S4 Table.

**Figure 6:**
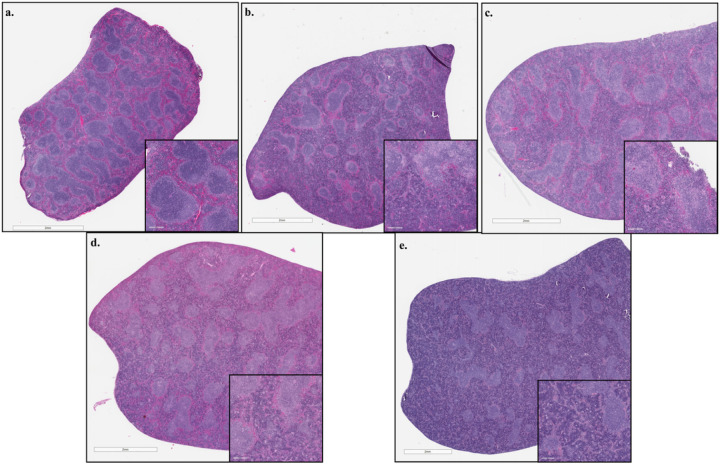
Spleen pathology Hematoxylin and eosin staining of spleen sections showed several histopathologic changes in infected mice. Control histology from an uninfected mouse (a) with normal structure. Reduction in average PALS diameter measured in HRTV (b) and HRTV + SGE (c) infected mice at 3 dpi. Increased hematopoiesis was observed in spleens of HRTV + SGE mice at 3 dpi (c), HRTV mice at 8 dpi (d), and HRTV + SGE (e) mice at 8 dpi. Depleted white pulp and absence of germinal centers (b—e) in infected groups at 3dpi and 8dpi. Comprehensive pathology observations and PALS averages for all mice are in S5 Table.

**Figure 7:**
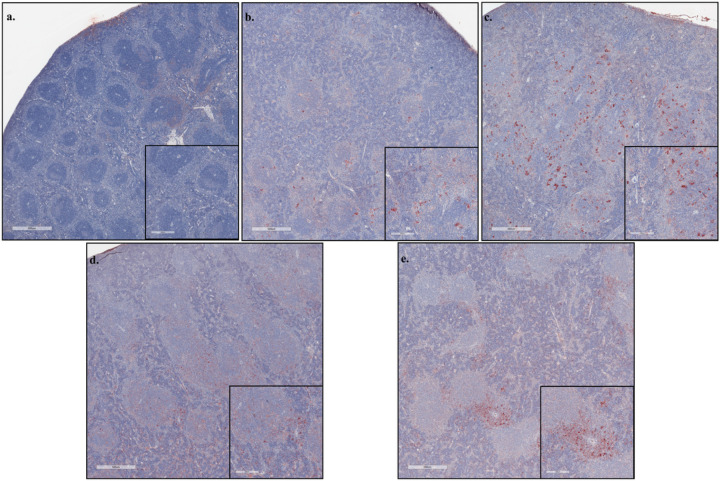
HRTV antigen detection in spleens Immunohistochemistry (IHC) was performed on spleen sections to visualize HRTV antigen (red signal) and counterstained with hematoxylin. Representative images of the uninfected spleen with minimal non-specific staining (a), HRTV infected mouse at 3dpi (b), HRTV + SGE infected mouse at 3dpi (c), HRTV infected mouse at 8dpi (d), and HRTV + SGE infected mouse at 8dpi (e). Scoring for antigen severity for all animals is located in S5 Table.

Pathological examination of H&E stained liver and spleen sections and spleen sections stained for viral antigen by IHC. The livers exhibited mixed inflammation of the portal tract and central vein present at 8 dpi and which was more severe in the HRTV + SGE group (Fig. 5, Supplementary Table S4). Megakaryocytes were observed at 3 and 8 dpi but seemed more prevalent in HRTV + SGE mice. Increased cellularity in sinusoidal spaces was observed in both groups at both timepoints, but the cell phenotype could not be determined. Granuloma-like lobular lesions with eosinophilic infiltrates were more prevalent at 3 dpi and in the HRTV-only mice. Spleens of all infected mice had extramedullary hematopoiesis (EH), depleted white pulp, and germinal centers were absent (Fig. 6, Supplementary Table S5). EH scores seemed slightly higher in the HRTV + SGE group at 3 dpi but were not different at 8 dpi. Both infected groups had slightly decreased average periarteriolar lymphoid sheath (PALS) diameter at 3 dpi. Uninfected control mice had an average PALS score of 307.79 (± 20.59), while HRTV-only mice were 280.75 (± 46.06), and HRTV + SGE mice were 282.98 (± 20.26). Viral antigen was most prevalent in the HRTV + SGE group at 3 dpi and was similar between infected groups at 8 dpi (Fig. 7, Supplementary Table S5). This was consistent with the viral load analysis for the spleens.

## Discussion

Tick-borne viruses are on the rise in the United States. Over the last few decades, there have been increases in the number of human cases of previously discovered and newly discovered viruses (3–11). The causes in the rise of tick-borne virus cases are not fully understood, but it has been suggested that increased awareness of these viruses and expanding geographic distribution of tick vectors are contributing factors. Heartland virus, first isolated in 2007, has been found in humans across 14 states in the Northeast, Midwest, and Southern United States (12–17, 35, 36). Seropositive wildlife and/or infected ticks have been found in areas that heavily overlap with the known range of *A. americanum* (20–23, 32–37). The current estimated range of the arthropod vector, *A. americanum*, includes regions where HRTV has not been found and is expected to continue expanding further north and west (27–31). This indicates there is potential HRTV infection across much of the United States. Furthermore, *Haemaphysalis longicornis*, the primary vector for Dabie bandavirus, was recently introduced to the United States and has been shown as a potential vector for HRTV under laboratory conditions (55).

The number of confirmed cases of HRTV is relatively low, and infected individuals have often had either or both a history of multiple tick bites and comorbidities (12). Combined with limited research conducted on HRTV, there are many unknown factors regarding the current and potential spread of the disease, transmission, and pathogenesis. Experimental transmission studies have shown that transstadial, vertical, and horizontal transmission of HRTV occurs in *A. americanum* under laboratory conditions, as well as seroconversion in a non-viremic host (39). This suggests that symptomatic HRTV infection in humans occurs only with significant exposure or when substantial comorbidities exist. Without additional studies identifying key risk factors for individuals who may be exposed to HRTV, the long-term effects of infection cannot be thoroughly evaluated.

During the development of animal models for arboviruses, it is imperative to evaluate interactions between pathogen, host, and vector as the vector feeding process has been shown to create an environment permissive to disease transmission and enhance severity via SAT (43, 47–49, 52). Evaluation of animal models for HRTV have demonstrated that IFN-deficient, but not immunocompetent, animals are susceptible to HRTV (38–42). In this work, we have shown that A129 mice develop a non-lethal disease course similar to human cases of HRTV, including splenomegaly, infection of multiple organs, and pathological changes in the liver and spleen. When HRTV is combined with *A. americanum* SGE, the disease severity and duration increase compared to infection with the virus alone. Utilizing the A129 mouse model for future evaluations may not only help in understanding HRTV transmission and pathogenesis but may also assist in determining the specific components of tick saliva which impact the course of the disease. Isolating these components could provide potential targets for developing vaccines to block disease transmission or potentially stop tick bites by preventing feeding.

As several tick species can transmit multiple pathogens, a vaccine that would prevent successful feeding has the potential to be much more effective than developing vaccines for individual pathogens but may be much more difficult to design. The composition of tick saliva has been shown to vary between tick species and life stages as well as change throughout the feeding process and in response to the host on which the tick is feeding (43–46, 48, 49, 56, 57). Therefore, identification of a single salivary factor across multiple species and life stages of a tick may not be possible. The changing composition of tick saliva throughout the feeding process presents an additional challenge to vaccine development. The duration of attachment required for *Ixodes scapularis* to transmit various pathogens has been fairly well studied, with some pathogens being transmitted as quickly as 15 minutes post-attachment. In contrast, other pathogens could take 24 to 48 hours (58–60). The minimum tick attachment time required for other tick species and pathogens, including *A. americanum* and HRTV, has been poorly studied and would require additional experiments to identify salivary factors presented early enough in the tick feeding process to prevent disease transmission.

## Supplementary Material

Supplement 1
