# Supplementary material for: The Lone Star Tick, Amblyomma americanum, salivary factors exacerbate the clinical outcome of Heartland virus disease in a small animal model
Source: Res Sq. 2023 Apr 25:rs.3.rs-2828801. Preprint. [Version 1] doi: 10.21203/rs.3.rs-2828801/v1 (PMC10168474; doi:10.21203/rs.3.rs-2828801/v1)
Supplement: Supplement 1 [file NIHPPRS2828801V1-supplement-1.pdf]

Supplementary Files

This is a list of supplementary files associated with this preprint. Click to download.

- SupplementaryTables.xlsx
